# Molecular Detection and Characterization of Hemotropic *Mycoplasma* in Assamese Macaques (*Macaca assamensis*) of Northern Thailand

**DOI:** 10.1155/2024/5539938

**Published:** 2024-05-13

**Authors:** Rucksak Rucksaken, Supakarn Kaewchot, Thitichai Jarudecha, Netnapa Vitithumakhun, Jira Niyomdham, Suchanit Ngamkala, Wanat Sricharern

**Affiliations:** ^1^Department of Veterinary Nursing, Faculty of Veterinary Technology, Kasetsart University, Bangkok, Thailand; ^2^Department of National Park, Wildlife and Plant Conservation, Ministry of Natural Resources and Environment, Bangkok, Thailand; ^3^Department of Parasitology, Faculty of Medicine, Kasetsart University, Bangkok, Thailand

## Abstract

Hemotropic mycoplasmas, also known as hemoplasmas, are parasitic bacteria that infect red blood cells, potentially leading to varying degrees of anemia across numerous mammalian species, including nonhuman primates. The present study aims to investigate the prevalence of hemoplasma infection and identify the species involved among free-ranging Assamese macaques (*Macaca assamensis*) inhabiting northern Thailand. A total of 133 blood samples were collected from Assamese macaques in Chiang Rai province, Thailand, and subjected to screening for hemoplasma infection utilizing nested PCR amplification targeting the 16S rRNA gene. Positive samples were subsequently analyzed through nucleotide sequencing and phylogenetic analysis for putative species identification. Current study results revealed that 17.3% (23/133; 95% CI 11.29-24.81) of Assamese macaques tested positive for hemoplasma infection using the nested PCR assay. Partial 16S rRNA sequences derived from hemoplasma isolates in Assamese macaques exhibited 99% homology, forming a cluster within the same phylogenetic clade as “*Candidatus* Mycoplasma haematomacacae,” previously identified in long-tailed macaques, rhesus macaques, and Japanese macaques. These findings suggest the presence of “*Ca*. M. haematomacacae” not only in long-tailed macaques and rhesus macaques but also in Assamese macaques in Thailand. To our knowledge, this marks the first molecular detection of “*Ca*. M. haematomacacae” in Assamese macaques in Thailand. These results hold significance as they enhance our understanding of hemoplasma infection distribution among macaque populations in Thailand.

## 1. Introduction

Hemotropic mycoplasmas (hemoplasmas) are Gram-negative, obligate red blood cell parasites that have not been successfully cultured. These parasitic bacteria simply adhere to the surface of red blood cells and do not penetrate the membrane [1]. Hemoplasmas are known to cause mild to life-threatening hemolytic anemia in various mammalian species, including dogs, cats, pigs, cattle [2], rodents [3], sheep, and goats [4]. The primary mode of transmission is believed to be through blood-sucking arthropods, but there is also a chance of direct transmission through aggressive cat interactions [5], and it appears that the primary method of transmission for hemoplasmas associated with rodents is through direct contact [6]. Although information regarding their vectors is still limited, hemoplasma DNA has been detected in other fleas from dogs and cats [7, 8], blood-sucking arthropods, including wild-caught mosquitoes [9], *Amblyomma* spp., and lice from rats [3]. Since hemoplasmas cannot be cultured in vitro, diagnostic methods have traditionally relied on blood smear examination and molecular diagnostic techniques such as PCR assays [1].

Hemoplasma infections have been reported in various nonhuman primate species, similar to other animals. Recently, molecular methods have detected at least three species of hemoplasmas in nonhuman primates. These species include “*Candidatus* Mycoplasma kahanei” in squirrel monkeys (*Saimiri sciureus*) [10], “*Candidatus* Mycoplasma aoti” in owl monkeys (*Aotus trivirgatus*) [11], and “*Candidatus* Mycoplasma haematomacacae” (formerly known as “*Candidatus* Mycoplasma haemomacaque” [12]) in cynomolgus monkeys (*Macaca fascicularis*) [13] as well as Japanese monkeys (*Macaca fuscata*) [14]. In Thailand, previous reports indicated “*Ca.* M. haematomacacae” infections in long-tailed macaques and rhesus macaques across several regions [15, 16].

The Assamese macaque (*Macaca assamensis*) is a species of macaque that can be found in Thailand. Some of these macaques are habituated to living in close proximity to humans and can be found in various locations, including the Wat Tham Pla temple in northern Thailand. Previous studies by Kaewpanus et al. have confirmed the presence of these macaques in Thailand [17, 18]. However, no reports have been made on the prevalence of hemoplasma infection in the Assamese macaques of Thailand. Therefore, the objective of this study was to determine the molecular prevalence and diversity of hemoplasmas in Assamese macaques from northern Thailand.

## 2. Materials and Methods

### 2.1. Ethical Statement

The present study adhered to the animal care and use guidelines established by the Ethical Review Board of the Office of the National Research Council of Thailand (NRCT) to ensure the ethical conduct of scientific research. The protocols and methods employed in this study underwent rigorous evaluation and were granted approval by the Kasetsart University Institutional Animal Care and Use Committee (approval number: ACKU59-VTN-004).

### 2.2. Blood Sample Collection and Study Site

**Figure 1:**
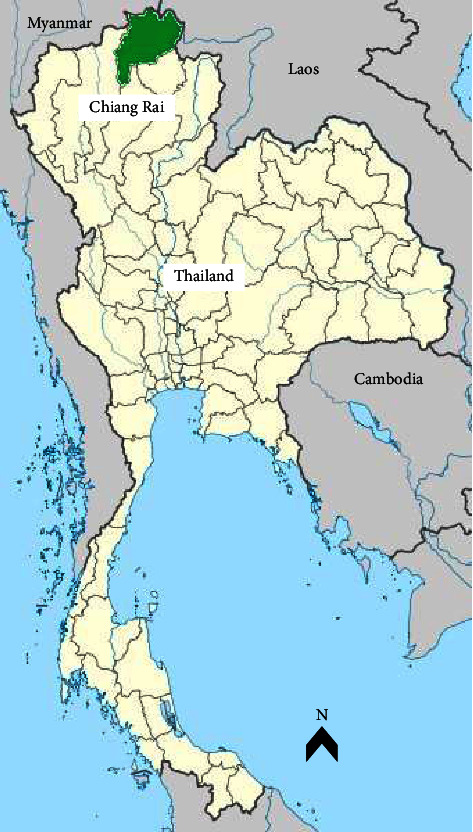
Sampling site locations in Chiang Rai province, northern Thailand (adapted from https://commons.wikimedia.org/wiki/File:Thailand_location_map.svg).

The study was carried out in July 2018 at Wat Tham Pla temple (Fish Cave Temple) (20°19′46.8″N 99°51′49.4″E), a renowned tourist attraction in Mae Sai district, Chiang Rai province, located in the northern region of Thailand (Figure 1). This specific location was selected due to its proximity to a thriving population of free-ranging Assamese macaques, coexisting with tourists and the local human community.

A total of 133 blood samples were collected from Assamese macaques using a convenience sampling method, comprising 98 males and 35 females. The macaques were trapped and anesthetized by veterinarians from the Department of National Parks, Wildlife, and Plant Conservation. Blood samples (up to 1 mL per animal) were then taken from femoral veins using a syringe and placed into EDTA tubes. To ensure preservation, all samples were kept cool with ice during transportation and subsequently stored at −40°C until further utilization for DNA extraction. Laboratory examinations were conducted at the Faculty of Veterinary Technology, Kasetsart University in Bangkok, Thailand.

### 2.3. Molecular Analysis

DNA extraction was performed on 250 *μ*L of each anticoagulated whole blood sample using an E.Z.N.A.® Blood DNA Mini Kit (OMEGA Bio-tek Inc.; Norcross, GA, USA), following the manufacturer's instructions. The concentration and quality of the extracted DNA were assessed using a Nanodrop (Thermo Scientific; Waltham, MA, USA), measuring the absorbance ratio between 260/280 nm. The extracted DNA was subsequently stored at −20°C until further analysis through nested PCR.

The amplification of the hemoplasma 16S rRNA gene was carried out using a broad-range nested PCR approach, employing primers previously described by Kaewmongkol et al. [17–19]. The primary PCR utilized V1-F and V9-R primers, while the secondary PCR employed V3-F and V6-R primers. The specific oligonucleotide sequences and thermal cycle conditions for both the primary and nested PCR are provided in Table 1. For each PCR reaction, a total volume of 20 *μ*L was used, comprising 1X PCR buffer, 2 mM MgCl2, 0.2 mM dNTPs, 1 *μ*M of each primer, and 0.04 U/*μ*L of Taq DNA polymerase (Invitrogen; Waltham, MA, USA). Two microliters of DNA extracted from the blood sample served as the template. To ensure accuracy and control for potential contamination, each PCR reaction included positive controls consisting of hemoplasma DNA from infected long-tailed macaques, confirmed by molecular analyses (submitted to the GenBank database under the accession numbers MZ960000 to MZ960022), as well as negative controls containing nuclease-free water.

The PCR products were subjected to identification through electrophoresis on a 1.2% (w/v) agarose-TAE gel, utilizing a 0.5X TAE buffer. The electrophoresis process was conducted at 100 V for a duration of 30 minutes. Following electrophoresis, the PCR products were stained using GelStar® (Cambrex Bio Science; Rockland, ME, USA) and visualized using ultraviolet transillumination. For the amplicons that exhibited the expected size, purification was performed, and they were subsequently analyzed using Sanger's sequencing technology by First BASE Laboratories (Sdn Bhd, Selangor, Malaysia).

### 2.4. Phylogenetic Analysis

Sequences obtained from positive samples in this study together with reference sequences downloaded from GenBank were aligned using the BioEdit program version 7.5.2. (https://bioedit.software.informer.com/). Phylogenetic analysis of the nucleotide sequences was created using the maximum likelihood method based on the Kimura 2-parameter model in MEGA-X software [20, 21]. A bootstrap analysis was used to assess the robustness of the clusters using 1,000 replicates.

### 2.5. Statistical Analysis

The data in this research were statistically analyzed using the STATA version 15.1 (Stata Corporation, Texas, USA). The statistical analyses were performed in order to evaluate associations and odd ratio (OR) between infection of hemoplasmas and sex of the Assamese macaques using univariable logistic regression. The analysis was conducted utilizing 95% confidence intervals (95% CI) determined through the following formula: 95% CI = $p\pm \left[1.96\times \sqrt{\left\{p\left(1-p\right)/n\right\}}\right]$. The results were considered significantly different when *p* < 0.05.

## 3. Results

The infection prevalence among male Assamese macaques was 20.4% (20/98; 95% CI 12.93–29.74), while that among females was 8.6% (3/35; 95% CI 1.80–23.06). Univariate logistic regression revealed that the odds ratio of hemoplasma infection in samples from male macaques did not have a difference with female macaques (OR = 2.73, 95% CI = 0.76–9.85, *p*=0.124) (Table 2).

Through a BLASTN analysis utilizing partial 16S rRNA sequences (700 bp), it was shown that all sequences from this study were found to be 99.7% to 99.8% homologous to previously published sequences of “*Ca*. M. haematomacacae” in the GenBank database. The nucleotide sequences of the 16S rRNA genes of hemoplasmas from Assamese macaques in Thailand were submitted to the GenBank database under the accession numbers MZ960000 to MZ960022.

**Figure 2:**
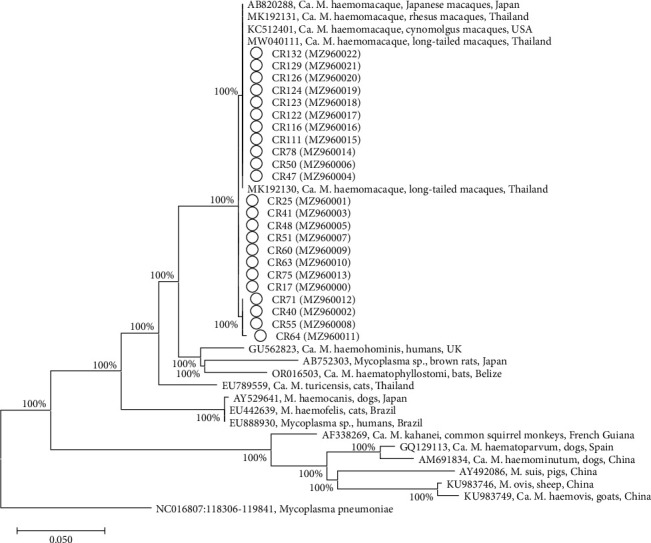
Phylogenetic tree based on 16S rRNA gene sequences of hemoplasma in Assamese macaques from Thailand (Ο) and other hemoplasma sequences from the GenBank database. GenBank accession numbers were shown for all sequences. The tree was constructed by the maximum likelihood method. *Mycoplasma pneumonia* strain 309 was used as the outgroup. The dataset was resampled 1,000 times to generate bootstrap percentage values which were shown at the nodes of the tree.

The phylogenetic tree analysis of 16S rRNA gene sequences detected in Assamese macaques (MZ960000 to MZ960022) and reference sequences from GenBank was run using the maximum likelihood method which revealed that all obtained sequences in the current study were classified in the same clade with “*Ca*. M. haematomacacae” detected in rhesus macaques (*M. mulatta*) and long-tailed macaques (*M. fascicularis*) from Thailand, cynomolgus macaques (*M. fascicularis*) from USA, and Japanese macaques (*M. fuscata*) from Japan (Figure 2).

## 4. Discussion

The current study detected evidence of “*Ca*. M. haematomacacae” in free-ranging Assamese macaques in Chiang Rai province in the north of Thailand which was similar with previous studies that detected “*Ca*. M. haematomacacae” in various species of nonhuman primates in many regions of Thailand including long-tailed macaques [15, 16] and rhesus macaques [15].

The prevalence of “*Ca*. M. haematomacacae” infection in Assamese macaques in the current study was quite low. Compared with other studies in nonhuman primates, there have been reports of 100% (9/9) in Japanese macaques from Japan [14], 84.6% (44/52) and 55.1% (125/227) in long-tailed macaques from the USA and Thailand, respectively [13, 15], and 65.7% (23/35) in rhesus macaques from Thailand [15]. However, the prevalence in this report was higher than another study in long-tailed macaques in Thailand (11.2%, 38/339) [16].

The current study also suggested that Assamese macaques in Chiang Rai province, Thailand, had a relatively high prevalence of “*Ca*. M. haematomacacae” infection, with male macaques being marginally more likely. However, the higher prevalence in male macaques was similar to other studies in various nonhuman primates from Brazil [22] and in long-tailed macaques from Thailand [16]. On the other hand, a higher prevalence in female macaques was reported in another study in long-tailed macaques from Thailand [15]. The odds ratio, which is a measure of the strength of association between two variables, was also calculated to compare the risk of infection between male and female macaques. The analysis found no significant difference in the odds of infection between males and females.

While human infections with hemoplasmas are rare, there have been documented cases and previous research has substantiated the existence of hemoplasma infections in various animal species capable of transmitting to humans. For instance, cases of *Mycoplasma haemofelis*-like infection have been reported among HIV-positive individuals in Brazil [23], and swine hemoplasmas have been detected in farmworkers in China [24]. Human hemoplasmosis may be asymptomatic or have various clinical signs similar to those seen in animals, including hemolytic anemia and pyrexia [25–27].

The phylogenetic findings suggested the occurrence of hemoplasma transmission among different macaque species within Thailand. Understanding the prevalence and transmission dynamics of these pathogens in macaque populations and other wildlife populations can have implications for public health [28]. The multilocus sequence analysis conducted in Thailand revealed a closer relationship of “*Ca*. M. haematomacacae” with hemotropic mycoplasmas found in capuchin monkeys, suggesting a possible origin from bats [29]. This result was consistent with the current study, as the phylogenetic analysis indicated that the hemoplasma identified in Assamese macaques was grouped within a clade closely related to the clade of hemoplasma strains found in bats from Belize [30]. Moreover, close contact between humans and macaques, particularly in areas where nonhuman primates living in free-ranging habitats near humans, could potentially increase the risk of zoonotic transmission [31]. Preventive measures to reduce the risk of hemoplasmosis in both monkeys and humans include controlling parasites, such as fleas and ticks, practicing good hygiene, and avoiding contact with infected blood or bodily fluids [32]. Additionally, prompt diagnosis and treatment of infected individuals are essential to prevent the spread of the disease.

## 5. Conclusion

In conclusion, to the best of our knowledge, this study represents the first investigation into the molecular detection of “*Ca*. M. haematomacacae” infection among free-ranging Assamese macaques in Thailand. Future research endeavors should encompass the examination of hemoplasmas in diverse nonhuman primate species across different geographical regions within Thailand, as well as among individuals inhabiting the same environments, to enhance our comprehension of the transmission dynamics of these parasitic bacteria to humans.

## Figures and Tables

**Table 1:** The oligonucleotide sequences, thermal cycling conditions used in the nested PCR assay for hemoplasma detection in Assamese macaques in Chiang Rai province, Thailand.

Primers	Oligonucleotide sequences (5′ ⟶ 3′)	Target gene	Thermal cycling conditions	Amplicon size (bp)
V1-F V9-R	AGAGTTTGATCCTGGCTCAG GNTACCTTGTTACGACTT	16S rRNA	95° (5 m); X40 [95° (1 m), 50° (1 m) and 72° (1 m)]; 72° (10 m)	1,400
V3-F V6-R	ACTCCTACGGGAGGCAGCAG CGACAGCCATGCANCACCT	16S rRNA	95° (5 m); X 45 [95° (1 m), 55° (45 s) and 72° (45 s)]; 72° (10 m)	700

**Table 2:** The prevalence and odds ratio of hemoplasma infection in Assamese macaques in Chiang Rai province, Thailand.

Sex	No. of animals	Positive *N* (%)	95% CI of proportion	OR	95% CI of OR	*P* value
Female	35	3 (8.6)	1.80–23.06			
Male	98	20 (20.4)	12.93–29.74	1	Reference	
Total	133	23 (17.3)	11.29–24.81	2.73	0.76–9.85	0.124

## Data Availability

The nucleotide sequences of the 16S rRNA genes of hemoplasmas from Assamese macaques from this study have been deposited in the GenBank database under the accession numbers MZ960000 to MZ960022. The datasets utilized and examined in the present study can be obtained from the corresponding author upon reasonable request.
